# Enhanced NETosis generation in radiographic axial spondyloarthritis: utility as biomarker for disease activity and anti-TNF-α therapy effectiveness

**DOI:** 10.1186/s12929-020-00634-1

**Published:** 2020-04-17

**Authors:** Patricia Ruiz-Limon, Maria Lourdes Ladehesa-Pineda, Maria del Carmen Castro-Villegas, Maria del Carmen Abalos-Aguilera, Clementina Lopez-Medina, Chary Lopez-Pedrera, Nuria Barbarroja, Daniel Espejo-Peralbo, Jose Antonio Gonzalez-Reyes, Jose Manuel Villalba, Carlos Perez-Sanchez, Alejandro Escudero-Contreras, Eduardo Collantes-Estevez, Pilar Font-Ugalde, Yolanda Jimenez-Gomez

**Affiliations:** 1 Instituto Maimónides de Investigación Biomédica de Córdoba (IMIBIC), Avda. Menéndez Pidal s/n, 14004 Córdoba, Spain; 2 Unidad de Gestión Clínica Reumatología, Hospital Universitario Reina Sofía, Avda. Menéndez Pidal s/n, 14004 Córdoba, Spain; 3 Departamento de Medicina (Medicina, Dermatología y Otorrinolaringología), Universidad de Córdoba, Avda. Menéndez Pidal s/n, 14004 Córdoba, Spain; 4 Unidad de Gestión Clínica Endocrinología y Nutrición, Instituto de Investigación Biomédica de Málaga (IBIMA), Hospital Clínico Virgen de la Victoria, Campus Teatinos s/n, 29010 Málaga, Spain; 5 Departamento de Biología Celular, Fisiología e Inmunología, Universidad de Córdoba, Campus de Excelencia Internacional Agroalimentario ceiA3, Campus de Rabanales, Edificio Severo Ochoa, 3ª planta, 14014 Córdoba, Spain

**Keywords:** Biomarkers/NETosis/neutrophils/immunotherapy/pathogenesis/radiographic axial spondyloarthritis

## Abstract

**Background:**

Radiographic axial spondyloarthritis (r-axSpA) is a chronic inflammatory form of arthritis in which tumor necrosis factor (TNF)-α, a potent inducer of inflammatory response and a key regulator of innate immunity and of Th1 immune responses, plays a central role. NETosis is a mechanism of innate immune defense that is involved in diverse rheumatology diseases. Nevertheless, spontaneous NETosis generation in r-axSpA, its association to disease pathogenesis, and the NETosis involvement on anti-TNF-α therapy’s effects has never been explored.

**Methods:**

Thirty r-axSpA patients and 32 healthy donors (HDs) were evaluated. Neutrophil extracellular trap (NET) formation, mediators of signal-transduction cascade required for NETosis induction and cell-free NETosis-derived products were quantified. An additional cohort of 15 r-axSpA patients treated with infliximab (IFX) for six months were further analyzed. In vitro studies were designed to assess the effects of IFX in NETosis generation and the inflammatory profile triggered.

**Results:**

Compared to HDs, neutrophils from r-axSpA patients displayed augmented spontaneous NET formation, elevated expression of NET-associated signaling components, nuclear peptidylarginine deiminase 4 translocation and increased citrullinated histone H3. Furthermore, patients exhibited altered circulating levels of cell-free NETosis-derived products (DNA, nucleosomes and elastase). Additional studies revealed that cell-free NETosis-derived products could be suitable biomarkers for distinguish r-axSpA patients from HDs. Correlation studies showed association between cell-free NETosis-derived products and clinical inflammatory parameters. Besides, nucleosomes displayed potential as a biomarker for discriminate patients according to disease activity.

IFX therapy promoted a reduction in both NETosis generation and disease activity in r-axSpA patients. Mechanistic in vitro studies further unveiled the relevance of IFX in reducing NET release and normalizing the augmented inflammatory activities promoted by NETs in mononuclear cells.

**Conclusions:**

This study reveals that NETosis is enhanced in r-axSpA patients and identifies the NETosis-derived products as potential disease activity biomarkers. In addition, the data suggests the potential role of NET generation analysis for assessment of therapeutic effectiveness in r-axSpA.

## Background

Spondyloarthritis (SpA) encompasses a group of chronic inflammatory rheumatic disorders that shares common clinical features, extra-articular manifestations and a strong genetic association with the human leukocyte antigen (HLA)-B27 [1]. The most frequent phenotype is the radiographic axial SpA (r-axSpA), which predominantly affects the axial skeleton and the sacroiliac joints [1]. This clinical form is more common in men than women (ratio 2:1), with a mean age of symptom onset around 26 years [2], and a prevalence ranges between 0.1 and 1.4% globally [3].

The pathological process of r-axSpA is characterized by an early inflammatory stage, followed by a phase of pathological new bone formation with syndesmophytes and ankylosis [4], which lead to a decrease in quality of life in these patients. Although an ever-growing list of factors and mechanisms seem to have a role in the pathogenesis of this disease [5–7], numerous gaps remain in our understanding of the precise molecular processes associated with the triggering, initiation, development, and regulation of this disease.

Immune system cells are essential in the r-axSpA pathogenesis, and although a considerable body of evidence exists regarding the central role of T lymphocytes in the pathological process [8–11], the role of neutrophils has not been examined to the same extent in this disease [12, 13]. Neutrophils are well established players in the immediate response to infections, employing a broad array of strategies including phagocytosis, release of toxic granular enzymes and of reactive oxygen species (ROS), or the generation of NETosis to remove harmful microorganisms [14, 15]. In the NETosis process, neutrophils expulse their nuclear contents to form neutrophil extracellular traps (NETs) that capture and kill microorganisms [16], and are composed of decondensed chromatin (neutrophil DNA and high affinity histones), which is covered with antimicrobial peptides and oxidant-generating enzymes [i.e. neutrophil elastase (NE), myeloperoxidase (MPO), NADPH oxidase, and nitric oxide synthase] [17–19]. The underlying signal-transducing pathway leading to NETs formation involves calcium mobilization, proinflammatory mediators, generation of ROS, nuclear delobulation by NE and MPO, and citrullination of histones by peptidylarginine deiminase 4 (PAD4), all of them essential to chromatin decondensation and DNA extrusion [18, 20–24].

Whereas the initial observation of NET formation showed to this process as an essential strategy to immobilize and kill invading microorganisms, extensive evidence suggest that aberrant NET formation is also central in a number of pathologies including rheumatoid arthritis (RA) and systemic lupus erythematosus (SLE) [25–27]. However, to date no study has evaluated the NET generation and the regulation of their release in SpA patients.

Therefore, the present study was designed to determine the NETotic response of neutrophils in r-axSpA, with particular concern to the underlying signal-transduction cascade, and whether the NETosis-derived products could be associated to r-axSpA, and could be useful for the prognostic and monitoring of the disease. In addition, as tumor necrosis factor (TNF)-α plays a central role in the pathogenesis of r-axSpA patients [28] and is a NETosis-inducing agent [24], the involvement of NETosis on the effects of infliximab (IFX), an anti-TNF-α drug, was further evaluated.

## Methods

### Study design and participants

In total, 30 r-axSpA patients and 32 healthy donors (HDs) matched for age and sex were included in the cross-sectional study. One more cohort of 15 r-axSpA patients treated with IFX (3 mg/kg/day intravenous infusion at times 0, 2 and 6 weeks, and every 8 weeks thereafter) for six months was also studied. All the r-axSpA patients fulfilled ASAS classification criteria for the classification of axSpA [29]. Exclusion criteria were: pregnancy, malignancies, chronic infections, other rheumatology diseases, extra-articular manifestations [psoriasis, inflammatory bowel disease (IBD), uveitis] and patients unable to understand the procedures to the protocol.

The study was conducted according to the principles of the Declaration of Helsinki. All experimental protocols were approved by the ethics committee of the Reina Sofía University Hospital, Córdoba, Spain, and written informed consent was obtained.

In the cross-sectional study, a case report form was used to collect the following clinical data: age, gender, disease duration, HLA-B27 status, plasma TNF-α and interleukin (IL)-1β concentration (pg/mL), and current medications. Disease activity was determined by C-reactive protein (CRP; nmol/L), erythrocyte sedimentation rate (ESR; mm/h) and the Bath Ankylosing Spondylitis Disease Activity Index (BASDAI) [30]. Spinal functionality and mobility of patients were evaluated by the Bath Ankylosing Spondylitis Functionality Index (BASFI) [31] and the Bath Ankylosing Spondylitis Metrology Index (BASMI) [32], respectively. Structural damage was assessed by the modified Stoke Ankylosing Spondylitis Spine Score (mSASSS) [33].

In the longitudinal study, the case report form included the following clinical data:

- Baseline: age, gender, disease duration, HLA-B27 status, and current medications. Disease activity was determined by CRP (mg/L), ESR (mm/h), and the BASDAI [30]. Functionality of r-axSpA patients were evaluated by the BASFI [31]. Structural damage was assessed by the mSASSS [33].
- After six months of IFX treatment: Disease activity was determined by CRP (nmol/L), ESR (mm/h), and the BASDAI [30]. Functionality of r-axSpA patients were evaluated by the BASFI [31].

### Blood sample collection and assessment of biological parameters

Peripheral venous blood samples were collected after a fasting period (8 h) in sterile tubes containing 0.129 M sodium citrate (Becton Dickinson, Meylan, France) and Z Serum Sep Clot Activator (Vacuette, Madrid, Spain), and were processed within 4 h of collection. Plasma and serum were aliquoted and stored at − 80 °C until their analysis. Laboratory markers of inflammation (ESR, CRP) and genetic factors (HLA-B27) were quantified as part of routine patient management.

### Inflammatory markers in plasma

Plasma levels of TNF-α and IL-1β were examined using a Bio-Plex Pro Assay (Bio-Rad Laboratories, Hercules, CA, USA), according to manufacturer’s instructions.

### White blood cells isolation

Neutrophils were isolated from patients and HDs’ blood by density centrifugation over Dextran-Ficoll as described by Nauseef et al [34]. As neutrophils could be activated by the isolation method, CD11b and CD62L expression were evaluated in healthy neutrophils (*n* = 5) from buffy coat (800 x g for 15 min at room temperature (RT) with the brake off centrifugation) and after dextran sedimentation followed Ficoll-Hypaque density gradient centrifugation. Both neutrophil activation markers were measured by flow cytometry (single-laser FACScalibur cytometer, BD Biosciences, San Jose, CA, USA) using anti-human CD11b PE (BD Biosciences) and anti-human CD62L FITC (eBioscience, San Diego, CA, USA). Non-statistically significant difference in CD11b and CD62L expression was found between buffy coat and the isolation method (Fig. S1).

Peripheral blood mononuclear cells (PBMCs) from HDs´ whole blood were separated by Ficoll gradient centrifugation (StemCell Technology, Oslo, Norway).

Purity of the populations was assessed by flow cytometry (single-laser FACScalibur cytometer, BD Biosciences) by analyzing the size and complexity (forward and size scatters). The purity was routinely ≥95% for both neutrophils and PBMCs.

### NETs generation and quantification

Neutrophils isolated from r-axSpA patients (*n* = 6) and HDs (*n* = 6) were seeded into 24-well plates (6 × 10^5^ cells per well) on poly-L lysine-coated glass coverslips (BD Biosciences) at 37 °C under 5% CO_2_. Then, cells were incubated in vitro for 6 h to determine spontaneous NETosis or treated with 600 nM of the potent NETosis inducer PMA (Sigma-Aldrich, San Luis, MO, USA) for 2 h. Next, neutrophils were fixed with 4% paraformaldehyde (15 min) and rinsed three times with PBS. Firstly, a Sytox orange dye staining (Life Technologies, Bleiswijk, Netherlands) (5 mM) was used to visualize DNA using a Nikon Eclipse-Ti-S fluorescence microscope (Nikon, Amsterdam, Netherlands). To evaluate NETosis generation, a total of five images selected randomly from different regions of each coverslip per case were taken with a 20x objective in each sample. NETs were manually identified on digitalized images as Sytox-positive structures emanating from cells with overall length greater than 2x cell area from cells untreated [35] and were counted for at least 3 times by two independent observers using IMAGE-J software (NIH, Bethesda, MD, USA). Results were expressed as percentage of NETs (NETs formation).

Secondly, neutrophils were stained with NE antibody and DAPI (as nuclear staining). Briefly, fixed cells were exposed to anti-human NE antibody (1:40) [RbmAb to Neutrophil Elastase (Abcam, Cambridge, UK)] overnight at 4 °C, and then incubated with Alexa-Fluor 488-conjugated secondary antibody (1:1000) (Abcam) for 1 h at 4 °C. Nuclear DNA was detected by incubating marked cells with DAPI (Invitrogen, Carlsbad, CA, USA) (300 nM) for 3 min at RT. Samples were visualized using a Nikon Eclipse-Ti-S fluorescence microscope (Nikon). The recorded images were loaded onto IMAGE-J software (NIH) for analysis and quantified by two independent observers. The colocalization between NE and DAPI was estimated by determining an overlapping pixel map of the two fluorescent channels. Netting neutrophils were considered those positive for both NE and DAPI. NETs were calculated as the average of cells stained with NE and DAPI [(at least 5 fields of each coverslip per case (× 20)] normalized to the total number of cells. Results were expressed as percentage of NE/total cell number.

For scanning electron microscopy, neutrophils from r-axSpA patients (*n* = 3) and HDs (*n* = 3) were fixed in 2.5% glutaraldehyde in 0.1 M cacodylate buffer (pH 7.2) for 45 min, washed twice in the same buffer for 10 min each and dehydrated in a series of ethanol. Absolute ethanol was changed 3 times (10 min each). Afterwards, ethanol was removed and the samples were kept at 35 °C for 20 min and gold shadowed using a High Vacuum Coater Leica EM ACE600 (Leica Microsystems, Barcelona, Spain). Samples were viewed and photographed using a JEOL JSM-7800F Field Emission Scanning Electron Microscope (Jeol, Tokyo, Japan).

### Quantification of supernatant cell-free DNA

Neutrophils isolated from r-axSpA patients (*n* = 6) and HDs (*n* = 6) were seeded with RPMI 1640 without phenol red (Sigma-Aldrich) into 96-well black microplate (2 × 10^4^ cells per well) and stained with 0.2 μM Sytox green dye (Sytox Green Nucleic Acid Stain, Invitrogen) for 6 h at 37 °C in the dark. As a positive control, neutrophils were incubated in the presence of 600 nM PMA (Sigma-Aldrich) for 2 h. Then, absorbance was read in an Infinite F200® Pro plate reader (Tecan Group Ltd., Männedorf, Switzerland) with a filter setting of 485 (excitation)/528 (emission). The results were expressed as relative fluorescence units (RFU) minus background control.

### Gene expression

Total RNA from neutrophils and PBMCs was extracted using TRIsure (Bioline, Taunton, MA, USA) following the manufacturer’s recommendations. A prerequisite for samples to be included in the RT-qPCR was a non-degraded pattern of the 18S and 28S subunits of ribosomal RNA in a 1.2% agarose gel. The RNA purity was verified by optical density (OD) absorption ratio OD260/OD280 that was between 1.8 and 2.0. Next, RT-qPCR was conducted in two steps. RT was performed using QuantiTect® Reverse Transcription kit (Qiagen, Hilden, Germany), following the manufacturer’s instructions. The expression levels of inflammatory mediators were then measured by real-time PCR using a LightCycler® Thermal Cycler System (Roche Diagnostics, Basel, Switzerland). The reaction was carried out with SYBR® Green PCR master mix (Takara Bio Inc., Madrid, Spain) according to manufacturer’s protocol. The primer sequences are listed in Table 1. GAPDH was selected as housekeeping gene. Real-time PCR data were analyzed by the 2^-ΔΔCt^ method. The expression of target genes was normalized to the mean of the housekeeping gene. All measurements were performed in duplicate. Controls consisting of reaction mixture without cDNA were negative in all runs. Fidelity of the PCR was determined by melting temperature analysis.

**Table 1:** Primer list

Primer	Supplier	Sequence 5′-3´	
		Forward	Reverse
**GADPH**	Sigma-Adrich, Steinheim, Germany	TGTAGTTGAGGTCAATGAAGGG	ACATCGCTCAGACACCATG
**STAT-3**	IDT, Leuven, Belgium	AGGCATTTGGCATCTGACAG	TGCTTCCCTGATTGTGACTG
**TNF-α**	IDT	TCAGCTTGAGGGTTTGCTAC	TGCACTTTGGAGTGATCGG
**IL-1β**	Qiagen, Hiden, Germany	Not provided by the manufacturer	Not provided by the manufacturer
**IL-1α**	IDT	AGTTCTTAGTGCCGTGAGTTTC	GTGACTGCCCAAGATGAAGA
**IL-6**	IDT	GCCCCACACAGACAGCCACTCACC	TGCCTCTTTGCTGCTTTCACACAT
**IL-23**	IDT	GGCGCAGAGCCAGCCAGATT	ACCCTCAGGCTGCAGGAGTTGG

GAPDH indicates glyceraldehyde-3-phosphate dehydrogenase; IL, interleukin; STAT, signal transducer and activator of transcription; TNF, tumor necrosis factor

### Determination of oxidative stress markers in neutrophils

Oxidative stress biomarkers were analysed in a single-laser FACSCalibur (BD Biosciences) cytometer. Test standardization and data acquisition analysis were performed using the CELL Quest software (BD Biosciences). A forward and side scatter gate was used for the selection and analysis of the different cell subpopulations.

For the assessment of ROS generation, including peroxides and peroxinitrites, cells were incubated with 20.5 μM Dichloro-dihydro-fluorescein diacetate (DCFHDA; Sigma-Aldrich) and 5 μM DihidroRhodamine-123 (DHRH123; Sigma-Aldrich) for 30 min in the dark at 37 °C. For the detection of intracellular glutathione (GSH), cells were incubated with 1 μM 5-chloromethylfluorescein diacetate (CMF-DA) (Invitrogen) for 30 min in the dark at 37 °C. The cells were washed, re-suspended in PBS, and then analysed on a single-laser FACSCalibur (BD Biosciences) cytometer. The JC1 Mitoscreen assay (BD Biosciences) was used (final concentration 2 μM) to assess mitochondrial membrane potential (ΔΨm) according to manufacturer’s instructions.

### Myeloperoxidase (MPO) and NE protein expression

Whole peripheral blood (100 μl) was incubated with 1 ml of lysis buffer (BD Pharm Lyse Lysing Buffer, BD Biosciences) for 10 min at RT in the dark. After centrifugation at 300 g for 5 min at 4 °C, cells were fixed and permeabilized with 250 μl of buffer (BD Cytofix/Cytoperm™ Fixation/Permeabilization solution Kit with BD Golgi Plug™, BD Biosciences) for 20 min at 4 °C, and then exposed to either FITC anti-human MPO antibody (BD Biosciences) or anti-human NE antibody (1/20) [(RbmAb to Neutrophil Elastase (Abcam)]. After incubation of cells with the primary antibodies for 20 min at 4 °C, an Alexa Fluor-488 conjugated secondary antibody (1:1000) (Abcam) was added for 30 min at 4 °C to analysis NE levels. IgG isotypes were used as negative controls. Cells were washed and acquired on the flow cytometer FACSCalibur (BD Biosciences).

### Protein extraction and western blotting

Cytoplasmic and nuclear extracts were prepared according to standard protocols [36] and the Bradford assay method (Bio-Rad) was used to determine protein concentration. Then, 20 μg of both cytoplasmic and nuclear extracts were separated by SDS-PAGE. Antibodies against PAD4 (1:1000; Abcam), citrullinated histone H3 (CitH3) (1:1000; Abcam) and nuclear factor (NF)-κB (1:500; Santa Cruz Biotechnology, Inc., Santa Cruz, CA, USA) were dispensed overnight (4 °C) and peroxidase-conjugated secondary antibody was administered for 1 h (RT). The immunoreaction was visualized using ECL plus (GE Healthcare, Buckinghamshire, UK). Stain-free technology (Bio-Rad) was used as loading protein control and densitometric analysis was performed with Image Lab software (Bio-Rad).

### Detection of circulating levels of cell-free DNA, nucleosomes and elastase

To quantify circulating cell-free DNA, 10 μL of plasma from r-axSpA patients and HDs was placed into 96-well black microplate and stained with 1 μM Sytox green dye (Sytox Green Nucleic Acid Stain, Invitrogen) in Tris-buffered saline [TBS (50 mM Tris-Cl, pH 7.5 and 150 mM NaCl)] at RT for 15 min in the dark. Next, plates were read in Infinite F200® Pro plate reader (Tecan Group Ltd) with a filter setting of 485 (excitation)/528 (emission). The data were analysed using a serial dilution of DNA from salmon as calibration curve.

Nucleosomes were measured by using the Human Cell Death Detection ELISAPLUS (Roche Diagnostics) according to the manufacturer’s instructions. Briefly, monoclonal antibodies against DNA (double and single strand) and histones (H1, H2A, H2B, H3 and H4) were used to detect mono- and oligo-nucleosomes in serum from r-axSpA patients and HDs. Quantification of nucleosomes was carried out by photometric determination of the absorbance at 405 nm (reference wavelength: 492 nm). Neutrophil elastase was measured by PMN Elastase Human ELISA kit (Abcam) in serum from r-axSpA patients and HDs following the manufacturer’s recommendations.

### In vitro studies

To assess the effects of IFX on NETosis generation, HD neutrophils (*n* = 3) were isolated and pretreated for 15 min at 4 °C with FCRII blocking Reagent (Miltenyi Biotec, Bergisch Gladbach, Germany). Then, neutrophils were washed with PBS, centrifuged at 300 g for 10 min, seeded in 24 well plates on poly-L lysine-coated glass coverslips (BD Biosciences) (6 × 10^5^ cells per well) in RPMI 1640 supplemented with 20% BSA (PanReacAppliChem, Barcelona, Spain), 2 mM L-glutamine (Biowest, Nuaillé, France) and 1% ZellShield (Minerva Biolabs, BMBH, Berlin, Germany) at 37 °C and 5% CO_2_. Next, neutrophils were treated with TNF-α (8 ng/mL; PrepoTech, Rocky Hill, NJ, USA), a potent NETosis inducer, in the presence or in the absence of IFX (100 mg/ml; Pfizer, NY, USA) for 2 h. NETotic response was evaluated as previously described.

To evaluate the effects of IFX on the proinflammatory profile of mononuclear cells promoted by NETs, PBMCs from previous HDs (*n* = 3) were seeded in 24-well ultra-low attachment plate (Sigma Aldrich) (1 × 10^6^ cells per well) and treated for 24 h with supernatants obtained from above-described in vitro experiments which were pre-incubated in the presence or absence of 15 U/ml DNAse I (NZYTech-Genes & Enzymes, Lisbon, Portugal) for 10 min. PBMCs were harvested for protein and RNA determination.

### Statistical analysis

Statistical analysis used SPSS statistical software, version 19.0 for WINDOWS (SPSS Inc., Chicago, IL, USA). A statistical power calculation in our cross-sectional study indicated that accepting an alpha risk of 0.05 in a two-sided test with 30 subjects in the r-axSpA group and 32 in the HD group, the statistical power in the nucleosome levels was 100% to recognize as statistically significant the difference of means (1.12 in the r-axSpA group and 0.24 in the HD group). For our longitudinal study, the statistical power calculation pointed out than accepting an alpha risk of 0.05 in a two-sided test with 15 r-axSpA patients, the statistical power in the nucleosome concentration was 92% to recognize as statistically significant the difference of means (2.39 in the r-axSpA group before treatment and 1.04 at 6 months of IFX therapy). Although we used the nucleosomes to set statistical power, because this cell-free NETosis-derived product was a potential biomarker for the prediction of activity in r-axSpA patients, we were equally interested in changes in the other variables of our study. The statistical power in the remaining parameters was at least 80%. The normal distribution of variables (Gaussian data) to characterize differences in the analyzed parameters was assessed using the Kolmogorov-Smirnow test. For continuous data, comparison among variables were performed using Independent Student’s tests or Paired Student’s tests for Gaussian data and using Mann-Whitney rank sum tests, Wilcoxon tests or Kruskal-Wallis H tests for non-normally distributed data. Categorical data were analyzed using the χ2 Test or Fisher’s Exact Probability Test, as appropriate. Multiple linear regression analysis was performed to exclude the influences of potential cofounding variables. Correlations were assessed by Spearman’s rank correlation.

Receiver operating characteristics (ROC) curves and the area under the curve (AUC) analysis were used to determine the sensitivity, specificity and corresponding cut-off values for each circulating cell-free NETosis-derived products in order to distinguish r-axSpA patients from HDs, and for discrimination of active vs. non-active r-axSpA patients. Active patients were considered those with a CRP level >  47.62 nmol/L, and/or patients with a BASDAI score ≥ 4 and ESR > 20 mm/h [37]. Logistic regression was used to develop composite panels of biomarkers to identify signatures that could distinguish r-axSpA from HDs with the greatest sensitivity and specificity. ROC analysis for composite panels was calculated. *P* < 0.05 was considered statistically significant.

## Results

### Study population

Clinical and laboratory parameters of the r-axSpA patients and the healthy age- and sex-matched donors included in the cross-sectional study are described in Table 2. Patients had an average age of 46.4 ± 13.4 years, with a disease duration of 13.2 ± 11.3 years and a mean score of total BASDAI and mSASSS of 4.4 ± 2.5 and 19.2 ± 21.1, respectively. Overall, patients exhibited greater mean levels of inflammatory markers (ESR, CRP, TNF-α, IL-1β) than HDs (*P* < 0.05), and a total of 86.7% of patients presented HLA-B27 antigen. There were no patients affected by anterior uveitis, IBD or psoriasis, although four of them had presented anterior uveitis in the past. Regarding treatment, 93.3% were taking non-steroidal anti-inflammatory drugs and 6.7% sulfasalazine.

**Table 2:** Clinical and Laboratory Parameters of the Radiographic Axial Spondyloarthritis Patients and the Healthy Donors

	r-axSpA patients (*n* = 30)	Healthy Donors (*n* = 32)	*P*
**Clinical parameters**			
Women/men, n/n	5/25	10/22	0.180
Age, y	46.40 ± 2.45	41.06 ± 1.77	0.080
BASDAI	4.42 ± 0.45	…	
BASFI	4.73 ± 0.61	…	
BASMI	3.22 ± 0.31	…	
mSASSS	19.16 ± 3.86	…	
Disease duration, y	13.23 ± 2.07	…	
Extra-articular manifestations			
➣ Uveitis	0/30	0/32	
➣ Psoriasis	0/30	0/32	
➣ IBD	0/30	0/32	
**Laboratory parameters**			
HLA-B27 (%)	26/30 (86.67%)	…	
CRP^†^, nmol/L	122.57 ± 31.38	13.62 ± 3.25	**< 0.001**
ESR^†^, mm/h	19.84 ± 4.09	7.48 ± 1.00	**0.006**
TNF-α, pg/mL	10.89 ± 1.82	4.02 ± 1.20	**0.021**
IL-1β, pg/mL	2.46 ± 0.22	1.57 ± 0.27	**0.047**
**Treatments**			
NSAIDs (%)	28/30 (93.33%)	…	
Sulfasalazine (%)	2/30 (6.67%)	…	

Values are presented as mean ± SEM. The data were analyzed using an Independent Samples t test or a Mann-Whitney U test to evaluate statistical significance between r-axSpA patients and healthy donors. ^†^Non-normally distributed data. BASDAI indicates Bath Ankylosing Spondylitis Disease Activity Index; BASFI, Bath Ankylosing Spondylitis Functionality Index; BASMI, Bath Ankylosing Spondylitis Metrology Index; CRP, C-reactive protein; ESR, erythrocyte sedimentation rate; HLA, human leukocyte antigen; IBD, inflammatory bowel disease; IL, interleukin; mSASSS, modified Stoke Ankylosing Spondylitis Spine Score; NSAIDs, non-steroidal anti-inflammatory drugs; r-axSpA, radiographic axial spondyloarthritis; TNF, tumor necrosis factor

### Enhanced NETs generation in r-axSpA patients

**Fig. 1:**
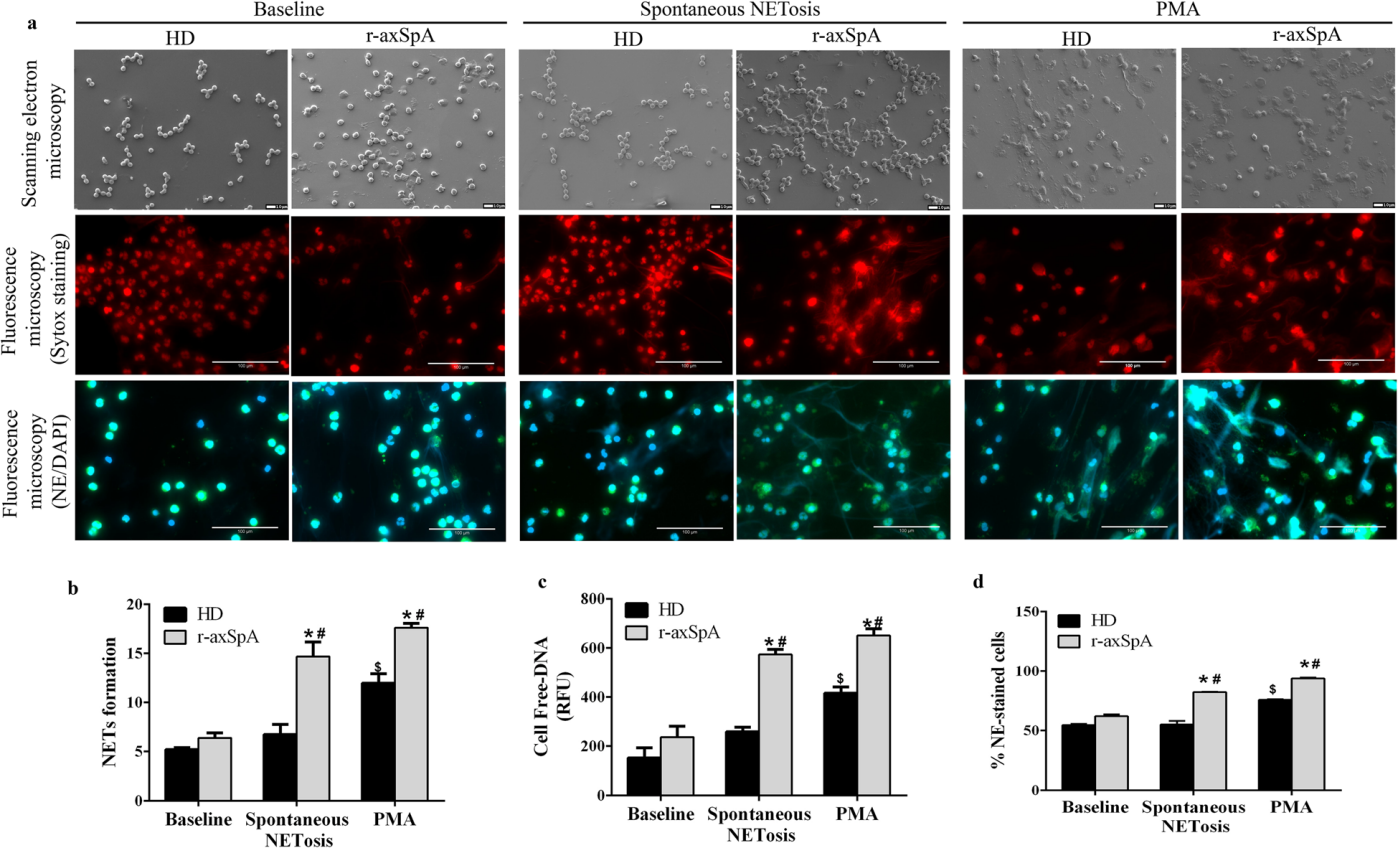
Enhanced NETosis generation in neutrophils from r-axSpA patients. (a-d) Neutrophils isolated from patients and healthy donors (HDs) were incubated ex vivo for 6 h -to determine spontaneous NETosis- or treated with or without PMA (600 nM) for 2 h (positive control). **a** Representative scanning electron micrographs of NETs from r-axSpA patients (*n* = 3) and HDs (*n* = 3) are shown in upper panels. Scale bar 10 μm. Fluorescence micrographs of NETs from patients (*n* = 6) and HDs (*n* = 6) stained with Sytox dye or with neutrophil elastase (NE) antibody and DAPI are represented in middle and lower panels, respectively. Images were captured at 20x magnification. Scale bar 100 μm. **b** NET formation. Results were expressed as percentage of NETs. **c** Supernatant cell-free DNA levels. Data are represented as relative fluorescence units (RFU). **d** % NE-staining cells. **b**-**d** Bar graphs show the mean ± SEM (HDs, *n* = 6; r-axSpA patients, *n* = 6). The data were analyzed using an Independent Samples *t* test or a paired-samples *t* test. *vs. corresponding HD control; ^#,$^vs. corresponding baseline (*P* < 0.05). NETs, neutrophil extracellular traps; r-axSpA, radiographic axial spondyloarthritis

Compared to HDs, r-axSpA-derived neutrophils displayed augmented spontaneous NETs extrusion, as demonstrated by fluorescence microscopy, fluorimetry, and scanning electron microscopy (Fig. 1a-d). Indeed, analysis of DNA fibers staining by SYTOX and cell-free DNA levels revealed that neutrophils from patients generated NETs to a greater magnitude than those from HDs after 6 h of ex vivo incubation (*P <* 0.05; Fig. 1a-c). Accordingly, statistically significant differences in DNA fibers and cell-free DNA levels were detected between both groups at 6 h (*P* < 0.05; Fig. a-c). Increased spontaneous NETs production in this pathology was additionally corroborated by the observation of an enhanced percentage of NE-staining cells at 6 h, as assessed by fluorescence microscopy (*P* < 0.05; Fig. 1a and d). PMA, known to promote NETosis, was used as positive control (Fig. 1a-d).

### Augmented expression of NET-associated signaling components, nuclear PAD4 translocation, and increased histone H3 citrullination in neutrophils from r-axSpA patients

**Fig. 2:**
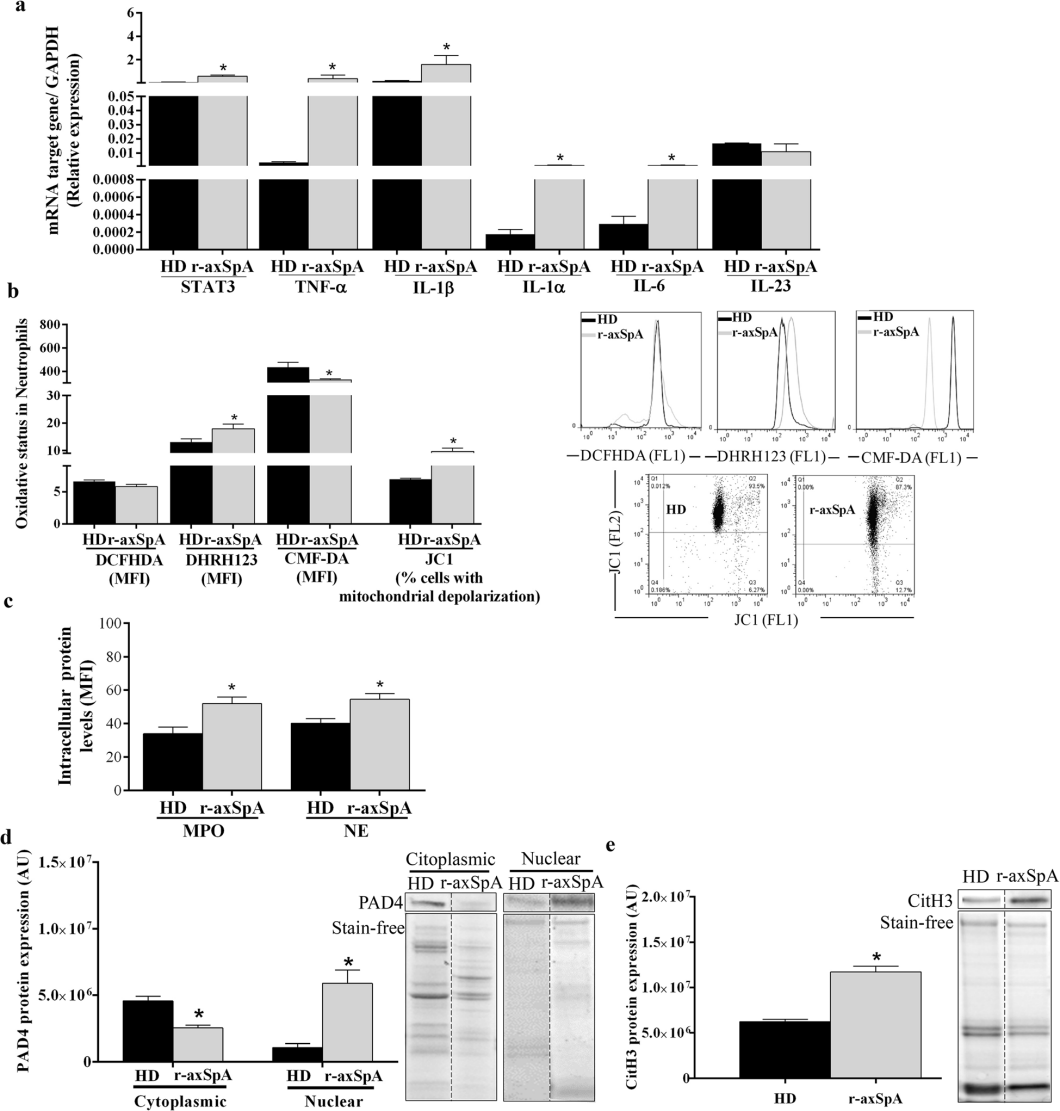
Neutrophils from r-axSpA patients display altered NETosis-related signal-transduction pathways. **a** Relative expression levels of inflammatory genes. **b** Peroxide and peroxinitrite production (DCFHDA and DHRH123), intracellular glutathione (CMF-DA) levels and the percentage of cells with mitochondrial depolarization. Bar graphs show the MFI to peroxide, peroxinitrite and glutathione levels. Representative histograms and dot plots are shown in right panels. **c** Intracellular MPO and NE protein expressions. Bar graphs show the MFI. **d** Relative PAD4 and CitH3 protein expression. Representative blots are shown. Lanes were run on the same gel but were non-contiguous. Cropping lines are used in the figure. **a**-**d** Values are presented as mean ± SEM (HDs, *n* = 32; r-axSpA patients, *n* = 30). The data were analyzed using an Independent Samples *t* test or a Mann-Whitney U test. *vs. HDs (*P* < 0.05). AU, arbitrary units; CitH3, citrullinated histone H3; HDs, healthy donors; IL, interleukin; MFI, mean fluorescence intensity; MPO, myeloperoxidase; NE, neutrophil elastase; PAD4, peptidylarginine deiminase 4; r-axSpA, radiographic axial spondyloarthritis; STAT, signal transducer and activator of transcription; TNF, tumor necrosis factor

We next studied signaling elements and molecular mechanisms involved in the NETosis process [21, 23, 24, 38]. In our hands, r-axSpA-derived neutrophils displayed augmented expression of signal transducer and activator of transcription 3 (STAT3), TNF-α, IL-1β, IL-1α and IL-6, as well as higher peroxinitrites production, reduced total GSH levels and increased percentage of cells with altered ΔΨm as compared to those from HDs (*P* < 0.05; Fig. 2a and b). Additionally, higher intracellular NE and MPO levels were found in r-axSpA-derived neutrophils as compared to HDs (*P <* 0.05; Fig. 2c).

Citrullination of histone subunits, such as H3, by PAD4 promotes chromatin decondensation [39]. Because PAD4 translocation to nucleus is necessary to exert its function, we next explored PAD4 localization (i.e. nucleus and cytoplasm) and observed that this enzyme was preferentially located in the nucleus of neutrophils from patients as compared to those from HDs (*P* < 0.05; Fig. 2d). The nuclear presence of PAD4 was associated with augmented CitH3 levels in neutrophils from r-axSpA patients vs. HDs (*P =* 0.016; Fig. 2e).

### The r-axSpA patients display altered levels of NETosis-derived products in circulation

**Fig. 3:**
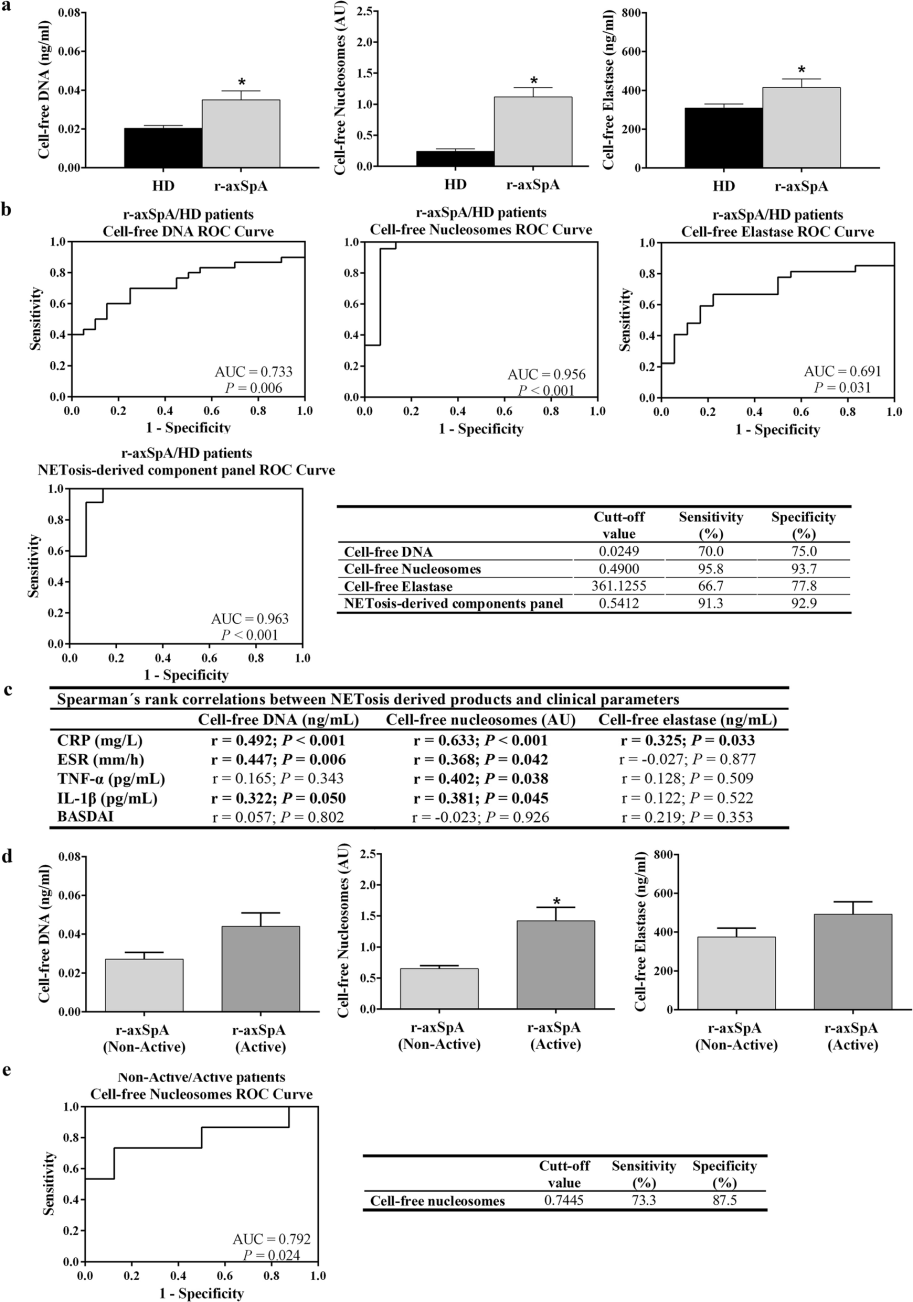
NETosis-derived products are augmented in r-axSpA patients and are biomarkers associated to r-axSpA and disease activity. **a** Circulating levels of NETosis-derived products. Values are represented as mean ± SEM (HDs, *n* = 32; r-axSpA patients, *n* = 30). The data were analyzed using an Independent Samples *t* test. *vs. HDs (*P* < 0.05). **b** ROC curve analyses of the NETosis-derived products and a combination of these as a panel were performed to assess their potential as biomarkers associated to r-axSpA. A cut-off value with higher specificity and sensitivity was tested. **c** Spearman’s rank correlations between NETosis-derived products and clinical inflammatory parameters. **d** Circulating cell-free NETosis-derived products in active (*n* = 18) and non-active r-axSpA patients (*n* = 12). Data are shown as mean ± SEM. The data were analyzed using an Independent Samples *t* test. *vs. HDs (*P* < 0.05). **e** A ROC curve analysis of the circulating cell-free nucleosomes was performed to assess its potential as biomarker for disease activity in r-axSpA. A cut-off value with higher specificity and sensitivity was tested. AU, arbitrary units; AUC, Area Under the Curve; BASDAI, Bath Ankylosing Spondylitis Disease Activity Index; CRP, C-reactive protein; ESR, erythrocyte sedimentation rate; HDs, healthy donors; IL, interleukin; r-axSpA, radiographic axial spondyloarthritis; ROC, receiver operating characteristics; TNF, tumor necrosis factor

Cell-free DNA levels were significantly higher in plasma samples from r-axSpA patients as compared to HDs (*P =* 0.007; Fig. 3a). In parallel, the concentrations of cell-free nucleosomes and elastase were also found elevated in r-axSpA serum related to HD serum (*P <* 0.05; Fig. 3a**)**. Next, multiple linear regression analysis was used to determine the association of these NETosis-derived products with age, gender and diagnosis (r-axSpA vs. HDs). No gender and age rather than diagnosis was statistically proven to act as a variable associated with cell-free DNA, nucleosome and elastase concentrations (Table S1). Next, r-axSpA patients were grouped in three blocks of disease duration (≤10 years, 11–20 years, > 20 years) in order to evaluate the association of this factor with the levels of these circulating cell-free NETosis markers. Comparisons among groups demonstrated non-significant difference in the concentration of these products (Table S2).

To study the relevance of the aforementioned NETosis-derived products as potential biomarkers of disease for r-axSpA patients, ROC curves analysis was carried out. As shown in Fig. 3b, circulating levels of cell-free DNA, nucleosomes and elastase could accurately distinguish r-axSpA patients from HDs, with the AUC ranging from 0.691 to 0.956. To further explore the accuracy of these circulating components for discriminating r-axSpA patients from HDs, a combination of the three-products as a panel was performed by using logistic regression on the data set. After multivariate analysis, no gender and age rather than diagnosis (r-axSpA vs. HDs) was statistically proven to act as a variable associated with the levels of the three-product signature (Table S1). Furthermore, comparative analysis between disease duration-stratified groups also showed non-significant difference in the levels of the NETosis-derived component panel (Table S2). The combination of these NETosis markers as a panel showed an evident accuracy, with an AUC of 0.963 (*P <* 0.001) at a sensitivity of 91.3% and a specificity of 92.9% from a cut-off value of 0.541 (Fig. 3b). Hence, NETosis-derived products may be suitable biomarkers for discrimination between r-axSpA patients and HDs.

### Circulating cell-free NETosis-derived products are associated to clinical inflammatory parameters

We next studied the correlation between circulating NETosis-derived products and clinical inflammatory parameters (Fig. 3c). Circulating cell-free DNA and nucleosome levels positively correlated with CRP, ESR and IL-1β concentrations. Besides, cell-free nucleosome levels were also positively associated with TNF-α concentration, and cell-free elastase levels with CRP concentration. Non-statistically significant correlation was observed between BASDAI and the NETosis-derived products.

### Utility of circulating cell-free nucleosomes as biomarkers for disease activity in r-axSpA patients

To further explore the potential usefulness of circulating NETosis-derived products as disease activity biomarkers, we analyzed the association of these NET components with the clinical activity of the r-axSpA patients. The patients were divided into two subgroups based on CRP and ESR levels, and the BASDAI score. Patients with a CRP level >  47.62 nmol/L and/or patients with a BASDAI score ≥ 4 and ESR > 20 mm/h were considered as active patients (*n* = 18), while the others were classified as non-active patients (*n* = 12) [37]. We found that cell-free nucleosome levels were significantly increased in active vs. non-active r-axSpA patients (*P =* 0.007; Fig. 3d). Non-statistically significant difference was detected in the rest of analyzed parameters.

To study whether cell-free nucleosomes can be used as potential biomarkers for disease activity in r-axSpA patients, ROC curve analyses were performed. This analysis revealed that nucleosomes displayed an evident accuracy, with a power AUC of 0.792 (*P* = 0.024; sensitivity = 73.3%, specificity = 87.5%, cut-off value = 0.744) (Fig. 3e). Consequently, our results suggest that circulating cell-free nucleosomes exhibits great potential as a biomarker for discrimination of active vs. non-active r-axSpA patients.

### Anti-TNF-α therapy reduces NETosis generation in r-axSpA patients

**Fig. 4:**
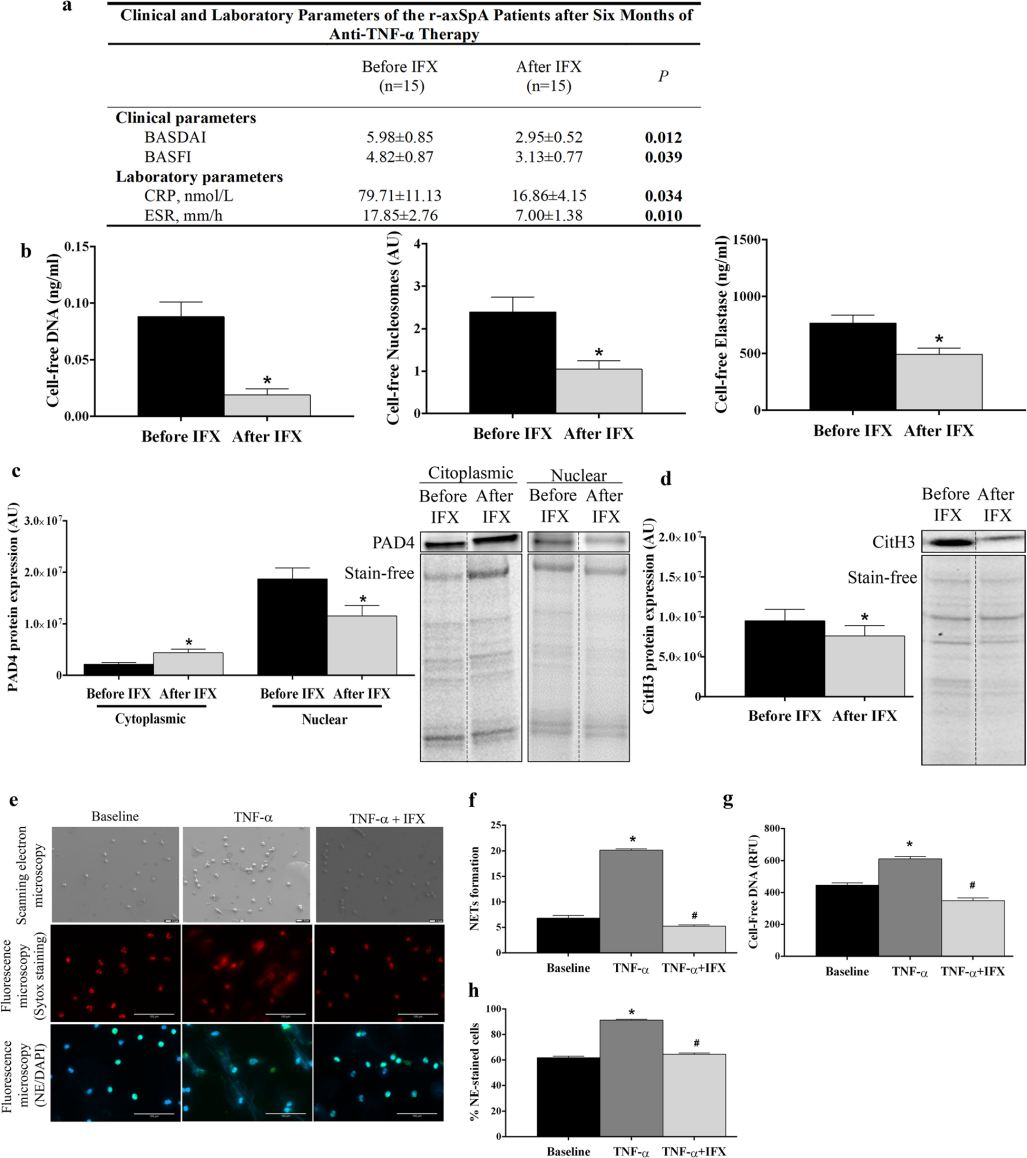
Anti-TNF-α therapy decreases NETosis. **a** Clinical and laboratory parameters, and (**b**) circulating levels of cell-free NETosis-derived products at baseline and after 6 months of infliximab (IFX) therapy. **c**-**d** Relative PAD4 and CitH3 protein expression at baseline and after IFX therapy. Representative blots are shown. Lanes were run on the same gel but non-contiguous. Cropping lines are used in the figure. **e**-**h** To assess the effect of IFX on NET formation, HD neutrophils (*n* = 3) were treated with TNF-α (8 ng/mL) in the presence or absence of IFX (100 mg/ml) for 2 h. **e** Representative scanning electron micrographs of NETs are shown in upper panels. Scale bar 10 μm. Fluorescence micrographs of NETs stained with Sytox dye or with neutrophil elastase (NE) antibody and DAPI are represented in middle and lower panels, respectively. Images were captured at 20x magnification. Scale bar 100 μm. **f** NET formation. Results were expressed as percentage of NETs. **g** Supernatant cell-free DNA levels. Data are represented as relative fluorescence units (RFU). **h** % NE-staining cells. **a**-**h** Bar graphs show the mean ± SEM. The data were analyzed using a paired-samples *t* test. *vs. baseline, #vs. TNF-α (*P* < 0.05). AU, arbitrary units; BASDAI indicates Bath Ankylosing Spondylitis Disease Activity Index; BASFI, Bath Ankylosing Spondylitis Functionality Index; CitH3, citrullinated histone H3; CRP, C-reactive protein; ESR, erythrocyte sedimentation rate; HD, healthy donor; NETs, neutrophil extracellular traps; PAD4, peptidylarginine deiminase 4; r-axSpA, radiographic axial spondyloarthritis; TNF, tumor necrosis factor

To explore the effect of anti-TNF-α therapy on expression levels of circulating components of NETs, a new cohort of 15 r-axSpA patients were included in a six-month longitudinal study (Table S3). After IFX therapy, the CRP and ESR levels, and the BASDAI and BASFI score improved significantly (*P* < 0.05; Fig. 4a). In parallel, r-axSpA patients exhibited a reduction in circulating levels of cell-free DNA, nucleosomes and elastase after six months of IFX treatment (*P* < 0.05; Fig. 4b). Accordingly, neutrophils displayed lower PAD4 expression levels in the nucleus compartment, along with a diminution of citH3 concentration after treatment (*P* < 0.05; Fig. 4c and d).

In vitro study showed that NETosis promoted by TNF-α was prevented by IFX, as demonstrated by a reduction in DNA fibers extrusion, cell-free DNA levels, and the percentage of NE-staining neutrophils (*P* < 0.05; Fig. 4e-h).

### Inhibition of NETs release by in vitro IFX treatment modulates the inflammatory profile in peripheral blood mononuclear cells

**Fig. 5:**
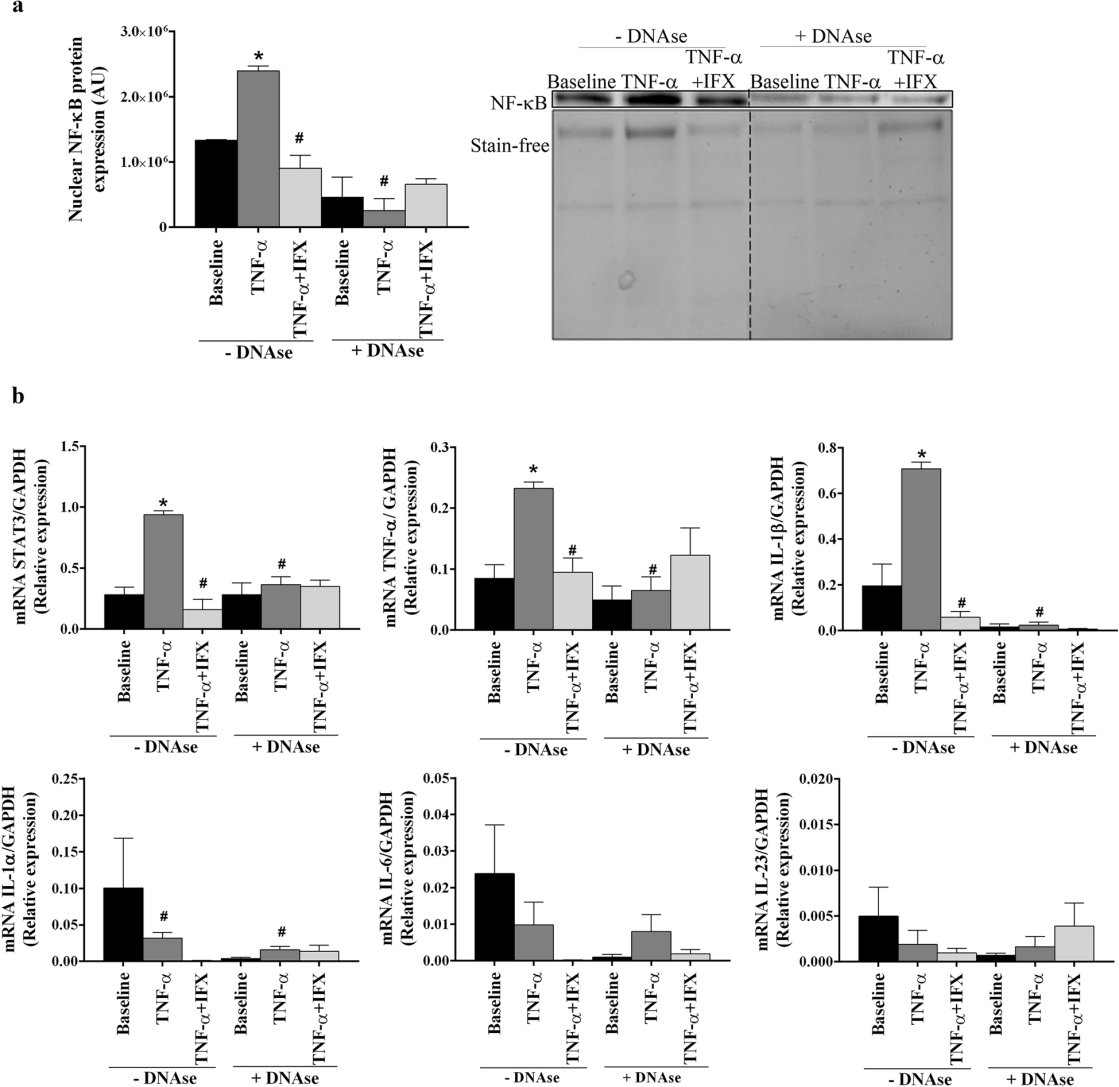
NET extrusion inhibition by in vitro IFX treatment reduces the inflammatory activity of mononuclear cells. To study the effects of infliximab (IFX) on the inflammatory profile of mononuclear cells promoted by NETs, HD neutrophils (*n* = 3) were treated with TNF-α (8 ng/mL) in the presence or in the absence of IFX (100 mg/ml) for 2 h. Then, supernatants, which were pre-incubated in the presence or absence of 15 U/ml DNAse I for 10 min, were added to PBMCs and further incubated for 24 h. **a** Nuclear NF-κB protein expression. Representative blot is shown. Lanes from different conditions were run on the same gel but were non-contiguous. Cropping lines are used in the figure. **b** Relative expression levels of inflammatory genes. **a**-**b** Bar graphs show the mean ± SEM. The data were analyzed using a paired-samples *t* test. *vs. baseline; ^#^vs. TNF-α (*P* < 0.05). AU, arbitrary units; HD, healthy donor; IL, interleukin; NET, neutrophil extracellular traps; NF-κB, nuclear factor-κB; PBMCs, peripheral blood mononuclear cells; STAT, signal transducer and activator of transcription; TNF, tumor necrosis factor

The prevention of NETosis generation by combined treatment of neutrophils with TNF-α plus IFX further promoted the normalization of inflammatory activities in PBMCs. Thus, while HD-derived neutrophils treated with TNF-α induced a translocation of NF-κB into the nucleus (*P* = 0.05; Fig. 5a) and the over-expression of proinflammatory mediators (*P* < 0.05; Fig. 5b) in PBMCs as compared to baseline, combined treatment with IFX significantly reduced those effects (*P* < 0.05; Fig. 5). The DNAse treatment abolished the TNF-induced increase of proinflammatory activities of mononuclear cells, indicating the NET process as the main inductor of circulating PBMCs. Hence, these data emphasize the role of NETosis on the establishment of an inflammatory status in r-axSpA patients, and the inhibitory effects of both of them by biological drugs.

## Discussion

To our knowledge, data from the current study were the first to show that r-axSpA-derived neutrophils are prone to generate spontaneous NETosis, underlying a new potential mechanism in the disease pathogenesis. In addition, we found that circulating cell-free NETosis-derived products, as biomarkers, could distinguish r-axSpA patients from HDs, and could discriminate patients according to disease activity. Besides, our study revealed a direct effect of anti-TNF-α therapy in inhibiting NETosis process, thus preventing the toxic side effects promoted by this phenomenon into inflammation.

The r-axSpA is a form of chronic multisystem inflammatory disorder [4], in which activated neutrophils play a crucial role in the progression of disease symptoms [13, 40]. Notwithstanding, to date, the potential involvement of NETotic events in the pathophysiology of this rheumatic disease has not been evaluated.

NETosis is a phenomenon involved in the innate immune response against infections by which neutrophils trap and/or kill pathogens. However, NET formation may function as a double-edged sword, contributing not only to pathogen control, but also as putative source of molecules with proinflammatory roles that may contribute to damage within inflamed tissues. Consequently, NETosis could be involved in the development and evolution of rheumatic diseases. In this regard, NET formation has been associated to the pathology of several autoimmune diseases, including RA and SLE [25–27]. The present study extends these observations and shows that NETosis is also enhanced in r-axSpA, further associated to changes in the underlying signal-transduction cascade required for the induction of this phenomenon. Among them, ROS generation is an essential process that induces NET formation. A previous study by Ugan et al., [12] demonstrated that r-axSpA-derived neutrophils displayed an oxidative status as compared to those from healthy controls. This observation was corroborated by our present findings, where an oxidative burden, evidenced by a disequilibrium between oxidant and antioxidant systems, and a significant loss in ΔΨm, was detected in r-axSpA-derived neutrophils. In addition, we extended these observations and found also elevations in other members of the NETosis-signaling pathway: r-axSpA-derived neutrophils displayed enhanced proinflammatory cytokine production, along with increased NE and MPO expression, nuclear translocation of PAD4, and citrullination of histone H3.Thereupon, these key elements required for efficient NET generation could further serve as potential targets for new therapeutic approaches.

Subsequently, we evaluated the potential of using NETosis-derived products as r-axSpA-related biomarkers. We found that circulating levels of cell-free DNA, nucleosomes and elastase were up-regulated in this disorder and that all of them had evident ability to distinguish between r-axSpA patients and HDs. The combination of the three NETotic markers as a panel also demonstrated to be a disease-related biomarker in r-axSpA. To additionally explore the relationship of these biomarkers with the pathology of r-axSpA, we next performed correlation studies, and confirmed that NETosis-derived products are related to clinical inflammatory parameters. Specifically, we found that levels of circulating cell-free NET components were significantly correlated with plasma cytokine levels (TNF-α, IL-1β), CRP and ESR. In addition, association studies demonstrated that circulating cell-free nucleosome levels were up-regulated in active vs. non-active r-axSpA patients. Moreover, the ROC curve analysis revealed that this component displayed value as biomarker for disease activity. These results are in concordance with a previous study performed by our group [25], where cell-free nucleosomes were showed to have diagnostic potential for discrimination of active vs. non-active RA patients.

The inhibition of NETosis in r-axSpA patients could be useful to avoid the deleterious effects of NETs on inflammation. In the context of r-axSpA, the introduction of TNF blocking drugs represented a therapeutic revolution for the pharmacological management of these patients, demonstrating to be highly effective in reducing systemic inflammation and improving the disease activity in these patients [41–43]. TNF is a cytokine that promotes inflammation, regulates the leukocyte extravasation, promotes tissue destruction and induces NET formation [24, 44]. Consequently, uncontrolled production or function of TNF has been associated to the pathology of several rheumatic diseases, including r-axSpA [45]. Recently, our research group evidenced that RA patients treated with IFX down-regulated NETosis generation in parallel to the reduction of both disease activity and the production of inflammatory mediators [25]. Based on these results and on the efficacy of anti-TNF-α drugs in r-axSpA, our present study evaluated the effects of IFX on both, NETosis generation and the toxic side effects promoted by NETs in inflammation. We found that IFX treatment not only promoted a reduction of disease activity but also a diminution NETosis generation in r-axSpA patients, evidenced by a down-regulation of circulating levels of NETosis-derived products, along with a lower expression of nuclear PAD4, and citH3 in r-axSpA-derived neutrophils. In vitro studies further unveiled the relevance of IFX in reducing NET release, as demonstrated by a decrease in DNA fibers, cell-free DNA levels, and the percentage of NE-staining neutrophils after this treatment. Besides, IFX therapy normalized the augmented inflammatory activities promoted by NETs in mononuclear cells. Additional analyses are required to elucidate the in vivo longer-term effects of IFX in the NETosis formation and the inflammatory profile triggered.

## Conclusions

Taken together, our data provide the first demonstration that NETosis is enhanced in r-axSpA patients and identify the potential role of circulating NETosis-derived products as novel complementary tools for discrimination of r-axSpA patients from HDs, and as key players for disease activity in this pathology. In addition, the present study establishes the inhibitory effect of anti-TNF-α therapy on NET formation, which parallels the reduction of disease activity. Thus, the analysis of NET generation might further have potential for the assessment of therapeutic effectiveness in r-axSpA patients.

## Supplementary information

**Additional file 1: Fig. S1.** shows CD11b and CD62L expression on healthy neutrophils from buffy coat and after density centrifugation over Dextran-Ficoll. **Table S1.** shows a multiple linear regression analysis on potential variables (i.e. age, gender and diagnosis) associated with NETosis-derived products. **Table S2.** shows the association of disease duration with the levels of circulating cell-free NETosis markers. **Table S3.** shows the clinical and laboratory parameters of the fifteen r-axSpA patients included in the longitudinal study.

## Data Availability

All supporting data have been shown in the current manuscript and its additional file.

## Abbreviations

AU: arbitrary units
AUC: area under the curve
BASDAI: Bath Ankylosing Spondylitis Disease Activity Index
BASFI: Bath Ankylosing Spondylitis Functionality Index
BASMI: Bath Ankylosing Spondylitis Metrology Index
citH3: citrullinated histone H3
CMF-DA: 5-chloromethylfluorescein diacetate
CRP: C-reactive protein
DCFHDA: dichloro-dihydro-fluorescein diacetate
DHRH123: dihidroRhodamine-123
ESR: erythrocyte sedimentation rate
GSH: glutathione
HDs: healthy donors
HLA: human leukocyte antigen
IBD: inflammatory bowel disease
IFX: infliximab
IL: interleukin
MFI: mean fluorescence intensity
MPO: myeloperoxidase
mSASSS: modified Stoke Ankylosing Spondylitis Spine Score
NE: neutrophil elastase
NETs: neutrophil extracellular traps
NF-κB: nuclear factor-κB
NSAIDs: non-steroidal anti-inflammatory drugs
OD: optical density
PAD4: peptidyl arginine deiminase 4
PBMCs: peripheral blood mononuclear cells
RA: rheumatoid arthritis
r-axSpA: radiographic axial spondyloarthritis
RFU: relative fluorescence units
ROC: receiver operating characteristics
ROS: reactive oxygen species
RT: room temperature
SLE: systemic lupus erythematosus
SpA: spondyloarthritis
STAT: signal transducer and activator of transcription
TNF: tumor necrosis factor
ΔΨm: mitochondrial membrane potential
