# Supplementary material for: Enhanced NETosis generation in radiographic axial spondyloarthritis: utility as biomarker for disease activity and anti-TNF-α therapy effectiveness
Source: J Biomed Sci. 2020 Apr 17;27:54. doi: 10.1186/s12929-020-00634-1 (PMC7164280; doi:10.1186/s12929-020-00634-1)
Supplement: Supplementary file 1 — Additional file 1: Fig. S1. shows CD11b and CD62L expression on healthy neutrophils from buffy coat and after density centrifugation over Dextran-Ficoll. Table S1. shows a multiple linear regression analysis on potential variables (i.e. age, gender and diagnosis) associated with NETosis-derived products. Table S2. shows the association of disease duration with the levels of circulating cell-free NETosis markers. Table S3. shows the clinical and laboratory parameters of the fifteen r-axSpA patients included in the longitudinal study. [file 12929_2020_634_MOESM1_ESM.docx]

**SUPPLEMENTARY INFORMATION**

**TITLE**: Enhanced NETosis generation in radiographic axial spondyloarthritis: utility as biomarker for disease activity and anti-TNF-α therapy effectiveness.

**AUTHORS**: Patricia Ruiz-Limon^1,2,3,4,*,†^, Maria Lourdes Ladehesa-Pineda^1,2,3,†^, Maria del Carmen Castro-Villegas^1,2,3^, Maria del Carmen Abalos-Aguilera^1,2,3^, Clementina Lopez-Medina^1,3^, Chary Lopez-Pedrera^1,2,3^, Nuria Barbarroja^1,2,3^, Daniel Espejo-Peralbo^1,2,3^, Jose Antonio Gonzalez-Reyes^5^, Jose Manuel Villalba^5^, Carlos Perez-Sanchez^1,2,3^, Alejandro Escudero-Contreras^1,2,3^, Eduardo Collantes-Estevez^3,2,1^, Pilar Font-Ugalde^1,3,2,†^, Yolanda Jimenez-Gomez, PhD^1,3,2,*,†^.

**AFFILIATIONS:** ^1^Instituto Maimónides de Investigación Biomédica de Córdoba (IMIBIC), Avda. Menéndez Pidal s/n, 14004, Córdoba, Spain; ^2^Unidad de Gestión Clínica Reumatología, Hospital Universitario Reina Sofía, Avda. Menéndez Pidal s/n, 14004, Córdoba, Spain; ^3^Departamento de Medicina (Medicina, Dermatología y Otorrinolaringología), Universidad de Córdoba, Avda. Menéndez Pidal s/n, 14004, Córdoba, Spain; ^4^Unidad de Gestión Clínica Endocrinología y Nutrición, Instituto de Investigación Biomédica de Málaga (IBIMA), Hospital Clínico Virgen de la Victoria, Campus Teatinos s/n, 29010, Málaga, Spain; ^5^Departamento de Biología Celular, Fisiología e Inmunología, Universidad de Córdoba, Campus de Excelencia Internacional Agroalimentario ceiA3, Campus de Rabanales, Edificio Severo Ochoa, 3ª planta, 14014, Córdoba, Spain.

†Patricia Ruiz-Limon, Maria Lourdes Ladehesa-Pineda, Pilar Font-Ugalde and

Yolanda Jimenez-Gomez contributed equally to this work.

*Corresponding authors.

**SUPPLEMENTAL FIGURE**

**
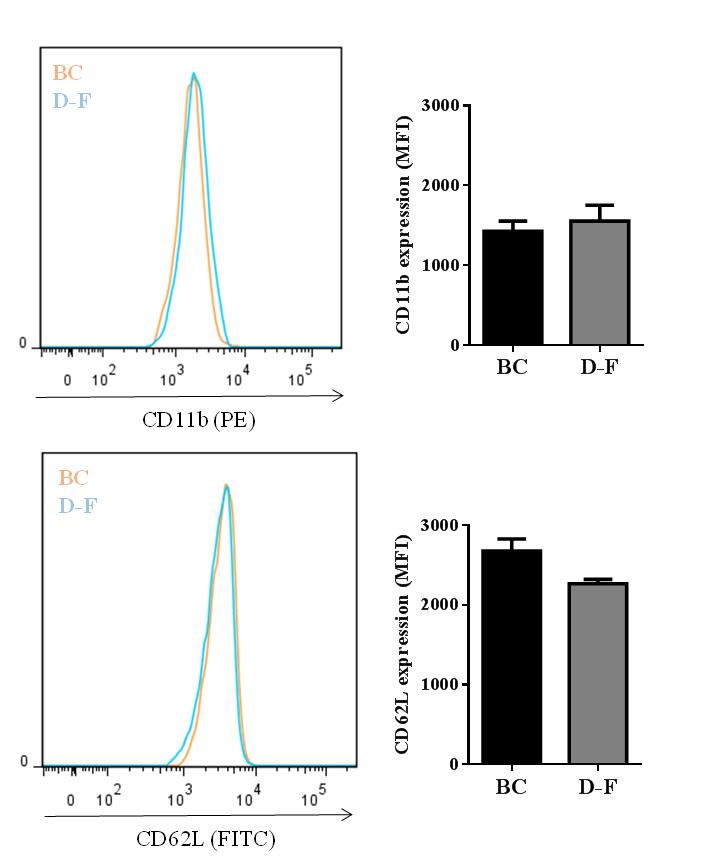
**

**a**

**b**

**Figure S1. CD11b and CD62L expression on healthy neutrophils from buffy coat and after density centrifugation over Dextran-Ficoll.** CD11b (a) and CD62L (b) expression levels on neutrophil cell surface (*n*=5). Representative histograms are shown in left panels. Data are displayed as mean fluorescence intensity (MFI). Bar graphs show the mean ± SEM. The data were analyzed using a paired-samples *t* test. BC, buffy coat; D-F, dextran sedimentation followed Ficoll-Hypaque density gradient centrifugation.

**SUPPLEMENTAL TABLES**

**Table S1. Multiple linear regression analysis on potential variables associated with NETosis-derived products.**

|  | **Independent**  **Variables** | **B** | ***P*** | **95% confidence intervals for B** | |
| --- | --- | --- | --- | --- | --- |
|  |  |  |  | **Lower boundary** | **Upper boundary** |
| **Cell-free DNA** | Model 1  Age  Gender  Diagnosis  Model 2  Gender  Diagnosis  Model 3  Diagnosis | 0.000  0.006  0.017  0.006  0.016  0.015 | 0.484  0.428  **0.013**  0.439  **0.016**  **0.021** | -0.001  -0.009  0.004  -0.009  0.003  0.002 | 0.000  0.020  0.030  0.020  0.028  0.027 |
| **Cell-free nucleosomes** | Model 1  Age  Gender  Diagnosis  Model 2  Gender  Diagnosis  Model 3  Diagnosis | 0.001  0.266  0.942  0.265  0.945  0.890 | 0.932  0.299  **<0.001**  0.293  **<0.001**  **<0.001** | -0-018  -0.246  0.582  -0.239  0.500  0.456 | 0.019  0.777  1.402  0.770  1.391  1.323 |
| **Cell-free elastase** | Model 1  Age  Gender  Diagnosis  Model 2  Gender  Diagnosis  Model 3  Diagnosis | -0.793  -36.359  142.286  -36.421  137.884  145.303 | 0.750  0.598  **0.030**  0.594  **0.029**  **0.018** | -5.777  -174.629  14.444  -173.107  14.501  26.183 | 4.191  101.910  270.129  100.265  261.266  264.423 |
| **NETosis-derived product panel** | Model 1  Age  Gender  Diagnosis  Model 2  Age  Diagnosis  Model 3  Diagnosis | -0.001  0.008  0.688  -0.001  0.686  0.683 | 0.840  0.929  **<0.001**  0.836  **<0.001**  **<0.001** | -0.007  -0.171  0.523  -0.007  0.529  0.531 | 0.006  0.186  0.854  0.006  0.844  0.836 |

**Table S2. Comparative analysis between disease duration-stratified r-axSpA groups.**

|  | **<10 years**  **(n=14)** | **11-20 years**  **(n=10)** | **>20 years**  **(n=6)** | ***P* value** |
| --- | --- | --- | --- | --- |
| **Cell-free DNA** | 0.032±0.008 | 0.037±0.009 | 0.040±0.013 | 0.618 |
| **Cell-free Nucleosomes** | 1.070±0.142 | 0.991±0.282 | 0.740±0.120 | 0.448 |
| **Cell-free Elastase** | 434.5±72.80 | 428.2±97.18 | 407.9±58.34 | 0.861 |
| **NEtosis-derived product panel** | 0.868±0.061 | 0.830±0.081 | 0.929±0.035 | 0.677 |

Values are presented as mean ± SEM. The data were analyzed using a Kruskal-Wallis H test to evaluate statistical significance between disease duration-stratified r-axSpA groups.

**Table S3. Clinical and laboratory parameters of the radiographic axial spondyloarthritis patients from longitudinal study.**

|  | r-axSpA Patients  (n=15) |
| --- | --- |
| **Clinical parameters** |  |
| Women/men, n/n | 2/13 |
| Age, y | 40.75±2.41 |
| BASDAI | 5.98±0.85 |
| BASFI | 4.82±0.87 |
| mSASSS | 14.87±6.39 |
| Disease duration, y | 11.87±2.25 |
| Extra-articular manifestations | |
| - Uveitis | 0/15 |
| - Psoriasis | 0/15 |
| - IBD | 0/15 |
| **Laboratory parameters** |  |
| HLA-B27 (%) | 15/15 (100%) |
| CRP, nmol/L | 79.71±11.14 |
| ESR, mm/h | 17.85±2.76 |
| **Treatments** |  |
| NSAIDs (%) | 15/15 (100%) |
| Methotrexate | 2/15 (13.33 %) |
| Sulfasalazine (%) | 4/15 (26.67%) |

Values are presented as mean ± SEM. BASDAI indicates Bath Ankylosing Spondylitis Disease Activity Index; BASFI, Bath Ankylosing Spondylitis Functionality Index; CRP, C-reactive protein; ESR, erythrocyte sedimentation rate; HLA, human leukocyte antigen; IBD, inflammatory bowel disease; mSASSS, modified Stoke Ankylosing Spondylitis Spine Score; NSAIDs, non-steroidal anti-inflammatory drugs; r-axSpA radiographic axial spondyloarthritis.
